# HPV Type Distribution in HIV Positive and Negative Women With or Without Cervical Dysplasia or Cancer in East Africa

**DOI:** 10.3389/fonc.2021.763717

**Published:** 2021-11-30

**Authors:** Ruby Mcharo, Tessa Lennemann, John France, Liseth Torres, Mercè Garí, Wilbert Mbuya, Wolfram Mwalongo, Anifrid Mahenge, Asli Bauer, Jonathan Mnkai, Laura Glasmeyer, Mona Judick, Matilda Paul, Nicolas Schroeder, Bareke Msomba, Magreth Sembo, Nhamo Chiwerengo, Michael Hoelscher, Otto Geisenberger, Ralph J. Lelle, Elmar Saathoff, Leonard Maboko, Mkunde Chachage, Arne Kroidl, Christof Geldmacher

**Affiliations:** ^1^ National Institute for Medical Research (NIMR)-Mbeya Medical Research Center (MMRC), Mbeya, Tanzania; ^2^ Division of Infectious Diseases and Tropical Medicine, University Hospital, LMU Munich, Munich, Germany; ^3^ Department of Obstetrics and Gynecology, Mbeya Zonal Referral Hospital, Mbeya, Tanzania; ^4^ Institute of Computational Biology, Helmholtz Zentrum München, Neuherberg, Germany; ^5^ Institute for Pathology, Otto-von-Guericke University Magdeburg, Magdeburg, Germany; ^6^ German Center for Infection Research (DZIF), Partner Site Munich, Munich, Germany; ^7^ Department of Gynecology and Obstetrics, University of Muenster, Muenster, Germany; ^8^ University of Dar es Salaam–Mbeya College of Health and Allied Sciences (UDSM-MCHAS), Mbeya, Tanzania

**Keywords:** human papilloma virus—HPV, human immunodeficiency virus—HIV, cervical cancer, cervical dysplasia, high-grade intraepithelial lesions, low-grade intraepithelial lesions, molecular diagnosis

## Abstract

**Background:**

Women living with HIV in sub-Saharan Africa are at increased risk to develop cervical cancer (CC), which is caused by persistent infection with 13 oncogenic human papilloma viruses (HR-HPVs). It is important to accurately identify and target HIV-positive women at highest risk to develop CC for early therapeutic intervention.

**Methods:**

A total of 2,134 HIV+ and HIV− women from South-West Tanzania were prospectively screened for cervical cancer and precancerous lesions. Women with cervical cancer (n=236), high- and low-grade squamous intraepithelial lesions (HSIL: n=68, LSIL: n=74), and without lesion (n=426) underwent high-resolution HPV genotyping.

**Results:**

Eighty percent of women who were diagnosed with HSIL or LSIL were living with HIV. Any lesion, young age, HIV status, and depleted CD4 T cell counts were independent risk factors for HPV infections, which were predominantly caused by HR-HPV types. While multiple HR-HPV type infections were predominant in HIV+ women with HSIL, single-type infections predominated in HIV+ CC cases (p=0.0006). HPV16, 18, and 45 accounted for 85% (68/80) and 75% (82/110) of HIV+ and HIV− CC cases, respectively. Of note, HPV35, the most frequent HPV type in HSIL-positive women living with HIV, was rarely detected as a single-type infection in HSIL and cancer cases.

**Conclusion:**

HPV16, 18, and 45 should receive special attention for molecular diagnostic algorithms during CC prevention programs for HIV+ women from sub-Saharan Africa. HPV35 may have a high potential to induce HSIL in women living with HIV, but less potential to cause cervical cancer in single-type infections.

## Introduction

Cervical cancer (CC) is the fourth most frequent cancer in women globally with 570,000 new cases and 311,000 deaths in 2018. Eighty-five percent of these CC cases occur in low- and middle-income countries (1). Sub-Saharan Africa (SSA) is the most heavily affected region globally; women here have the highest cumulative lifetime risk to develop CC—above 5% for many countries—and CC is the leading cause of death from malignancy in many SSA countries (2). These “quasi epidemic” dimensions at least partially result from regional particularities, including a poor screening capacity for identification of women at risk for CC development and high prevalence of HIV infection.

Thirteen “high risk” (HR) HPV types can cause CC and precancerous cervical intraepithelial neoplasias (CIN) within the anogenital region and the oral cavity (3, 4) and therefore have been classified as direct carcinogens. E6 and E7 oncoproteins from high-risk HPVs contribute to this carcinogenic potential and are able to drive cell cycle entry in the upper epithelial layers and to stimulate cell proliferation in the basal and parabasal layers (5). HPV16 (>50%) and 18 (>10%) account for the majority of CC cases worldwide, followed by HPV45 and HPV31, while the rest is caused by other high-risk HPV types (6, 7). HIV infection is a major risk factor to develop CC and other HPV-associated cancers. Two large American population-based studies in HIV-infected women reported a 9- and 5-fold increased risk to develop CC compared to HIV negative women (8, 9). Newly acquired HIV infection immediately increases the risk for detectable HR-HPV infections (10, 11), whereas chronic HIV infection is linked to a high prevalence of diverse HR-HPV type infections, longer HPV persistence, high risk for precancerous lesions, and a more rapid disease progression to CC (9, 12–17). HIV^+^ women from SSA are also affected by high prevalence rates of diverse oncogenic HR-HPV infections and associated reproductive tract cytologic abnormalities (16, 18–20). Sahasrabuddhe and colleagues found premalignant high-grade squamous intraepithelial lesions (HSIL) or squamous cell carcinoma (SCC) in more than half of HIV^+^ Zambian women seeking HIV/AIDS treatment (16), and these diagnoses were often associated with HPV types other than 16 and 18. This study and other studies support the hypothesis that HR-HPV types other than 16 and 18 more frequently cause CC in HIV^+^ compared to HIV^−^ women (15, 16, 21).

Diagnostic algorithms tailored to a specific SSA context can contribute to better guide CC prevention and therapeutic intervention strategies, such as cryotherapy and excision surgery, and guide design of therapeutic vaccines as a possible non-invasive alternative for women with persistent HR-HPV infection and premalignant lesions. Because of the apparent interaction of infections with HIV and carcinogenic HPVs, it is also important to accurately identify and target HIV+ women at highest risk to develop CC for early therapeutic intervention.

In order to identify risk factors for HR-HPV infection and to address the hypothesis that HR-HPV types other than HPV16 and 18 more frequently cause cancer in HIV+ *versus* HIV− women, we have studied HPV infection in 2H study participants with CC, high- and low-grade squamous intraepithelial lesions (HSIL and LSIL), and without cervical lesions in relation to HIV status, ART treatment, and disease progression. HSIL, CIN2 and 3 are referred to as HSIL in the manuscript. LSIL and CIN1 are referred to as LSIL in the manuscript.

## Material and Methods

### Study Settings and Participants

The 2H study is a prospective study in the Mbeya Region, South-West Tanzania, embedded within the Tanzanian cervical cancer screening program. Between March 2013 and August 2020, overall 2,146 women were included and screened for cytohistological diagnosis of precancerous and cancerous lesions. The majority of study participants were recruited in the cervical cancer screening program at the Department of Obstetrics and Gynecology and at the HIV care and treatment center (CTC) of the Mbeya Zonal Referral Hospital (MZRH). In addition, women were recruited from other CTC in the Mbeya region, during mobile CC screening activities using a Mobile Diagnostic and Testing Centre, and during CC mass community screenings conducted in Mbeya region. From these locations, women with positive visual inspection using acetic acid (VIA) findings or cancer-suspicious were specifically targeted for study inclusion. For study inclusion, women had to be 18 years and above. Exclusion criteria included current pregnancy, prisoners, mentally disturbed women, or women in a serious health condition for whom study participation or informed consent procedures would imply an undue burden. All study participants were fully briefed on the study procedures, and written informed consent was required prior to enrolment. Ethical clearance was obtained from the Mbeya Medical Research and Ethics Review Committee (MRH/R.10/8/Vol. VI/107), the Tanzanian National Health Research Ethics Committee (NIMR/HQ/R.8a/Vol. IX/1422), and the Ethics Committee of the Medical Faculty of Munich University before commencement of the study.

### Clinical Procedures

CC screening was performed following the Tanzanian National Guidelines by trained and certified nurses. All women underwent gynecological examination including speculum examination of the naive vagina and cervix uteri. The cervix was described for the clinical presence of cervicitis, tumor, ulceration, or other significant lesions. In addition to routine procedures, a Papanicolaou (pap) smear was taken from all women from the ectocervix using an Ayres spatula and two endocervical brush samples, one for cytology and one for HPV genotyping. In cases suspicious for cancer, biopsies were collected. Women diagnosed with CC or precancerous lesions (CIN2/CIN3) were referred to the Mbeya Zonal Referral Hospital (MZRH) gynecological clinic for re-assessment of lesions, and if indicated for FIGO staging. Treatment recommendation including Loop Electrosurgical Excision Procedure (LEEP), hysterectomy, or referral to the Ocean Road Cancer Institute in Dar es Salaam for radio-chemotherapy were provided by gynecologists.

### Evaluation of Histological and Cytological Pathology

Routine cytology by Pap testing was performed on all subjects for assessment of cervical pathology by two pathologists. If needed, further histologic characterizations were performed based on hematoxylin and eosin staining at the MZRH pathology department. The Bethesda system for reporting cervical or vaginal cytological diagnoses was used for reporting results. In addition, histological changes were described by degree of severity [No lesion, cervical intraepithelial neoplasia (CIN) 1, CIN 2, CIN 3, Cervical Cancer] if performed. For external quality control, a subset of cytology and histology slides was subjected to assessment by a third pathologist.

### Assessment of HIV-Associated Parameters

HIV status was determined for those with unknown HIV status. HIV history information, including data on antiretroviral therapy, were obtained through patient interviews, patient’s clinic cards, or extracted from hospital charts where applicable. For women with unknown or previously negative HIV status, HIV counseling and testing were performed based on the national HIV testing algorithm with a first screening rapid test (Determine HIV1/2, Abbott Laboratories, South Africa) followed by a second confirmatory rapid test (Uni-Gold HIV Rapid Test, Trinity Biotech, South Africa) if the first test result was positive. In the case of discordant rapid test results, the test was repeated with a different sample of the same patient. If the repeated test was still inconclusive, then an ELISA was performed (Bio-Rad GS HIV-1/HIV-2 PLUS O EIA, Bio-Rad laboratories GmbH, Munich, Germany). All tests were performed at the College of American Pathologists (CAP)-accredited MMRC laboratories.

### Detection of Human Papilloma Virus Infection and HPV Genotyping Procedures

**Figure 1:**
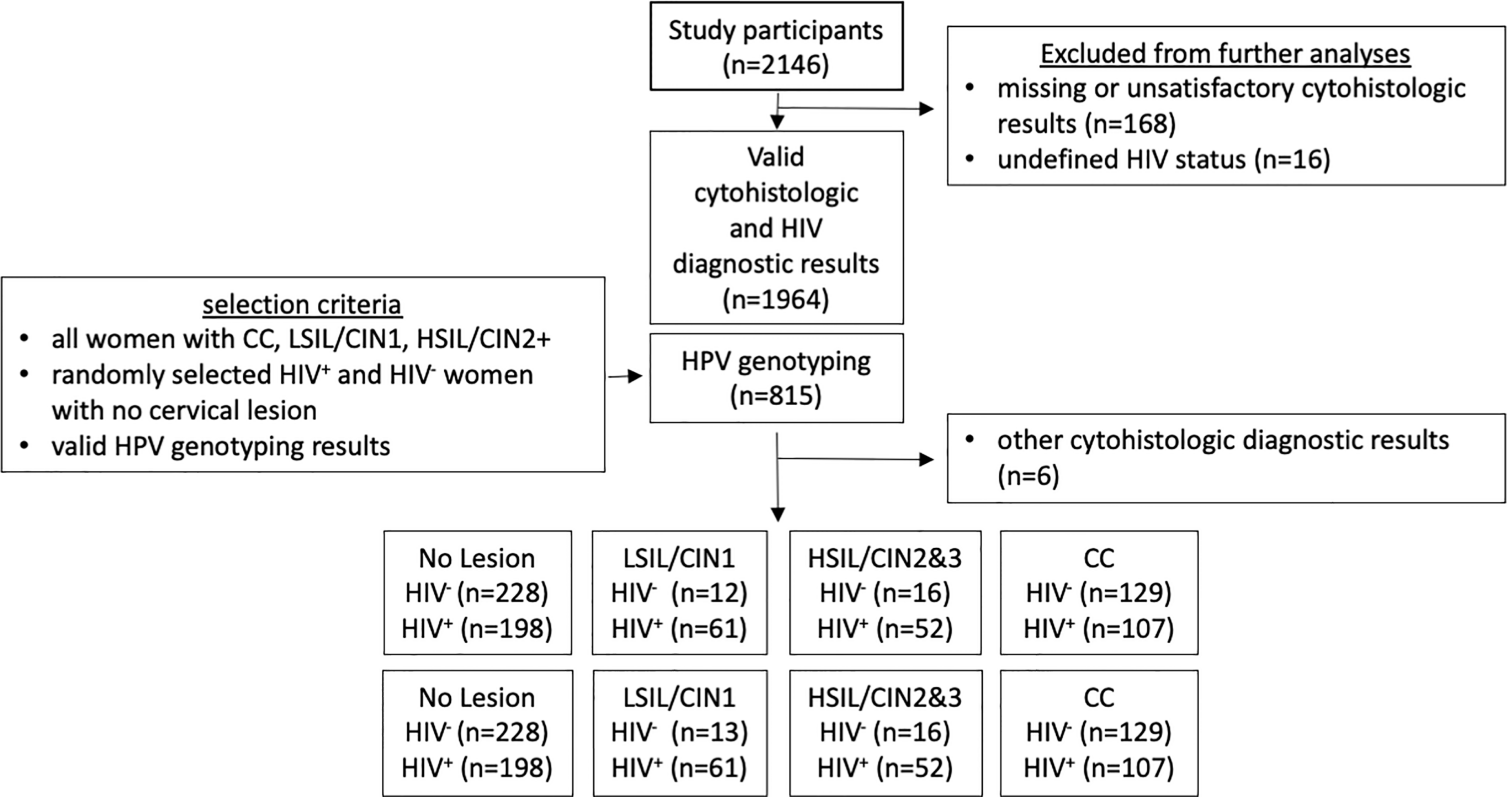
Flow Diagram of subjects included for HPV genotyping analyses. A total of 2,146 women were screened during the 2H study, including patient referrals with cervical cancer and women with a positive visual inspection with acetic acid result during cervical cancer screening program. Valid cytohistologic diagnoses based on Pap smear and/or histology was obtained for 1,964 of these women. HPV genotyping was then performed for 815 women and to include all cervical cancer cases, all women with cervical intraepithelial lesions, as well as the similar number of women living with or without HIV and with no lesions.

HPV genotyping was performed for selected participants with well-defined HIV status and cytohistological diagnosis as shown in **Figure 1**. Cervical cells collected by cytobrush sampling were transferred into medium (PreservCyt^®^ Solution) and sent to the molecular diagnostics laboratory at the NIMR-MMRC for HPV genotyping. HPV genotyping analysis was performed using the Linear Array^®^ HPV Genotyping Test (Roche Molecular Systems) according to manufacturer instructions. The test identifies 37 different HPV genotypes (6, 11, 16, 18, 26, 31, 33, 35, 39, 40, 42, 45, 51, 52, 53, 54, 55, 56, 58, 59, 61, 62, 64, 66, 67, 68, 69, 70, 71, 72, 73, 81, 82, 83, 84, IS39, and CP6108). Samples without detectable cellular DNA were excluded from analyses. Please note that this assay cannot distinguish HPV52 infections in samples containing HPV33, HPV35, and/or HPV58. Since 2013, the NIMR-MMRC HPV genotyping laboratory has regularly participated in the WHO HPV genotyping proficiency testing.

### Statistical Analyses

Data analysis and graphics were performed using the statistical software GraphPad prism 6.0 and R (R Development Core Team, 2021) and the ggplot R-package (22). For descriptive analyses, boxplots and scatterplots were used. Mann-Whitney (Wilcoxon) test was used to assess differences between groups in terms of age and cytohistological diagnosis stratified by HIV status. The two-sided Fisher’s exact test was used to determine whether the frequency of occurrence of individual HPV types significantly differs between HIV+ women with LSIL, HSIL, and CC being compared to HIV+ women without cervical lesion. Different multivariate regression models were used in order to assess the relationship between the number of HPV infections and related risk factors. The first model was performed on all the study participants and was adjusted for age in years, HIV status, and cytohistological diagnosis. The second model was performed on HIV+ women and was adjusted for age and cytohistological diagnosis, as well as antiretroviral treatment (ARV) and number of CD4 cells counts (below or above 250). And finally, a third model was applied on HIV+ women and adjusted for age and type of lesion (cancer *vs.* no cancer). P-values <0.05 were considered statistically significant.

## Results

### Cohort Characteristics

Between March 2013 and August 2020, 2,146 women from Mbeya region, South-West Tanzania, were enrolled into the 2H study. Of these women, 168 had missing diagnostic or unsatisfactory results. Other missing diagnostic included an undefined or inconclusive HIV status (n=16), as well as an undefined ART-status. All these women were excluded from analysis (**Figure 1**). Valid cytohistological and HIV diagnostic results were available for 1,964 of these women with a valid cytohistologic (Pap smear and/or histologic analyses). Other identified cervical pathologies included ASC-US (n=1), AGC Neoplasia (n=2), carcinoid (n=1), and tuberculosis of the cervix (n=2) and were also excluded from this analysis.

High-resolution HPV genotyping was performed on selected study participants to study characteristics of HPV infection in HIV+ and HIV− women in a case-control design, as shown in **Figure 1**. These analyses included valid results from 815 women; all cases of CC (n=236), all cases with HSIL or CIN2/CIN3 (HSIL, n=68), women with LSIL or CIN1 (LSIL, n=74) with available cytobrush samples, and a similar number of randomly assigned HIV− and HIV+ women without cervical lesions (n= 228 *vs* n=198, total 426). Of note, despite screening of more than 2,000 women during the study, only few HIV− women with HSIL or LSIL could be identified and included in these analyses. Indeed, 76% of HSIL cases and 84% of LSIL cases identified during the study period were women living with HIV. **Table 1** summarizes age and HIV disease progression parameters (ART status, HIV viral load and CD4 counts) for the study groups.

**Table 1:** Summarizes the age and—for HIV-positive women—ART status, viremic suppression, and CD4 counts.

Diagnosis	HIV− women		HIV+ women				
	N	Age (median, IQR)	n	Age (median, IQR)	On ART (%)	HIV-RNA <1,000 copies/ml (%)*	CD4 count (median, IQR)
No lesion	228	38 (32–48)	198	37 (31–42)	74% (NA: n=13)	62% (48/77)	407 (262–608)
LSIL	13	38 (27–52)	61	34 (29–40)	73% (NA: n=6)	64% (14/22)	387 (185–548)
HSIL	16	40 (36–51)	52	38 (32–43)	81% (NA: n=3)	56% (18/32)	368 (232–494)
Cancer	129	58 (48–74)	107	37 (39–50)	77% (NA: n=15)	68% (45/66)	358 (195–579)

*The chosen viral load cutoff is aligned with WHO guidelines for virological failure defined as two sequential viral loads (VL) levels of 1,000 or more copies/ml within 3 months (Organization 2016).

### Risk Factors for HPV Infection

**Figure 2:**
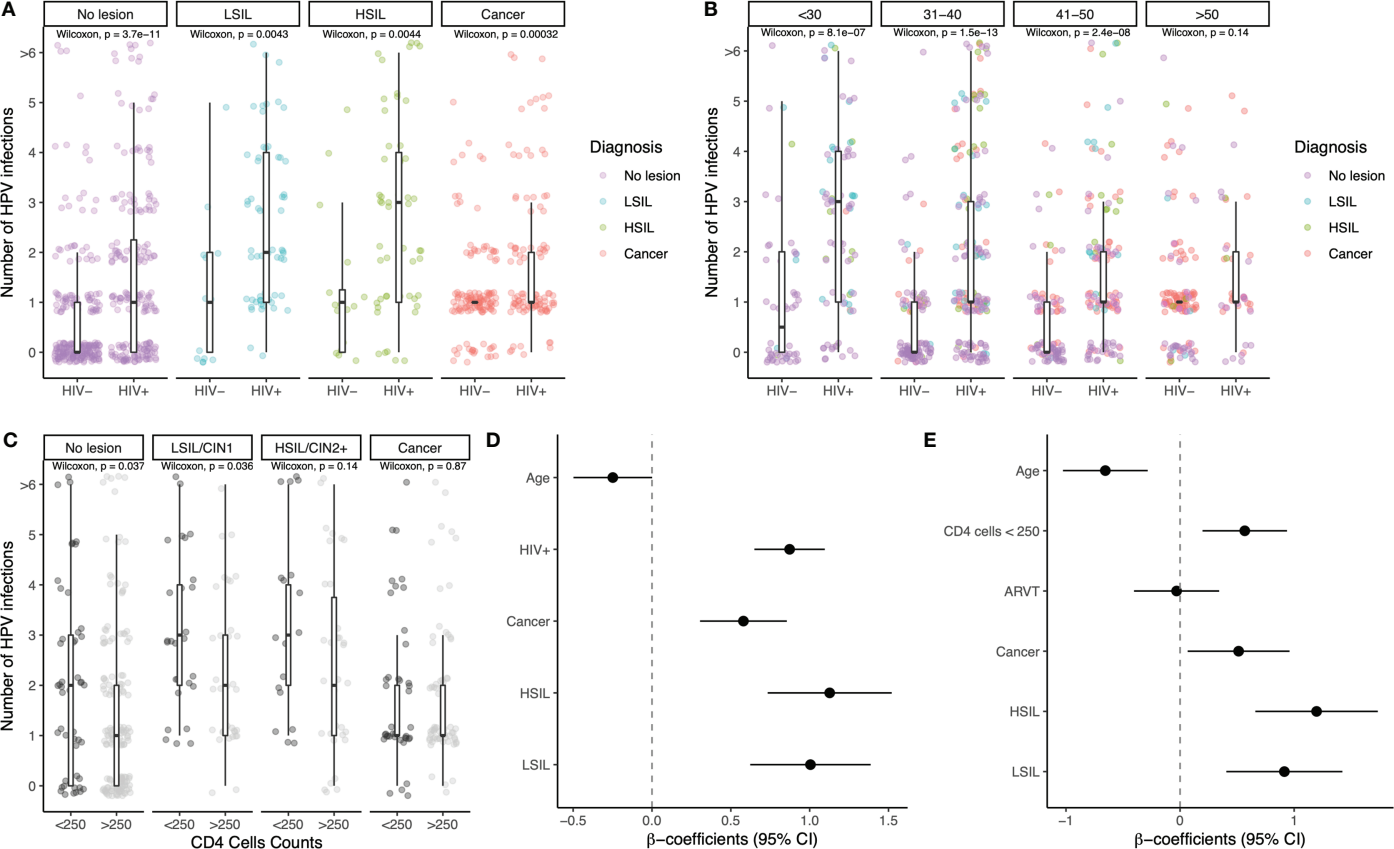
Number of different HPV infections in relation to suspected risk factors. Number of HPV infections stratified by HIV status and cervical lesion status **(A)**, by HIV status and age groups **(B)**, and for women living with HIV stratified by CD4 T cell count (below and above 250) and lesion status **(C)**. Boxplots showing the median and interquartile range as well as outliers are indicated. Statistical analyses were performed using the Wilcoxon test. Multivariate linear regression results with number of HPV infections as dependent variable are shown in **(D)** for all women, adjusting by age, HIV status, and cytohistological diagnosis, and **(E)** for women living with HIV, adjusting by age in years, cytohistological diagnosis, number of CD4 T cell count (above *vs.* below 250), and antiretroviral treatment (no *vs.* yes). The individual risk factors are indicated on the y-axis; beta-coefficients and 95% confidence intervals are shown on the x-axis. In order to better visualize different data points that overlap, we used jitter, a graphical representation strategy in the ggplot R-package.

Among the study participants with complete data, we determined the number of HPV types and stratified the results by HIV status and cytohistological diagnosis (**Figure 2A**). HIV infection was associated with a significantly higher number of detected HPV types in women regardless of cervical lesion or cancer status (p<0.005 for all). The proportion of HIV+ women (of all women) with multiple concurrent HPV-type infections was always higher, as compared to HIV-negative women (**Supplementary Figure 1**). HIV infection was also significantly associated with a higher number of different HPV-type infections in age groups up to 50 years of age (p<0.0001 for all age groups, **Figure 2B**). Within women living with HIV, more advanced immunosuppression defined by CD4 T cell counts of below 250/ul correlated with higher numbers of detected HPV types, particularly for those without lesions and those with LSIL (p<0.05), but not for those with cervical cancer (p=0.87), (**Figure 2C**).

The multivariate regression models showed similar trends. When including all the study participants, age was found to be negatively associated with number of HPV infections (beta-coefficient: −0.26, 95% CI: −0.51; −0.010), while HIV infection and the three cytohistological diagnoses of LSIL, HSIL, and Cancer were positively associated, with statistically significant results (**Figure 2D**). When the model was performed only on HIV+ women, the previous associations for age and cytohistological diagnosis remained, suggesting eventual clearance of most HPV infections over time even in HIV+ women. For participants with CD4 cell counts below 250, the association was found to be positive and statistically significant (beta-coefficient: 0.57, 95% CI: 0.20; 0.94), while ART was not found to be associated with the number of HPV infections (beta-coefficient: −0.031, 95% CI: −0.40; 0.34) (**Figure 2E**).

### Characterization of HPV Genotypes in Groups Classified by HIV Status and Cytohistologic Diagnosis

**Figure 3:**
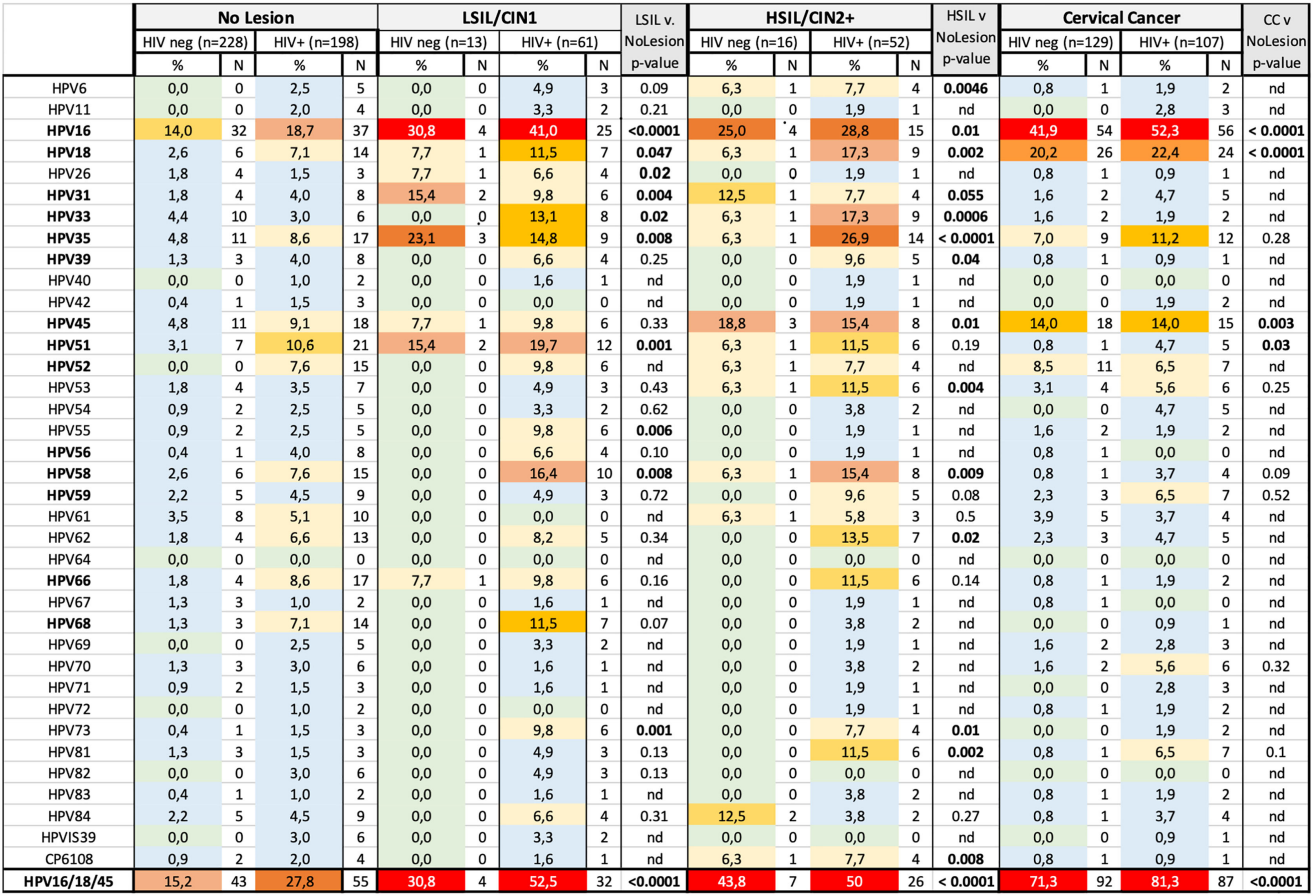
Frequency of occurrence of HPV types in HIV+ and HIV− women stratified by cytohistological diagnosis. HR-HPV types are indicated in bold. Frequencies are color coded in different shades as follows: 0% (light green), 0.1 to 5.0% (light blue), 5.1 to 10.0% (light yellow), 10.1 to 15.0% (light orange), 15.1 to 20.0% (faint red), 20.1 to 25.0% (orange), 25.1 to 30.0% (red-orange), >30.0 (strong red). The two-sided Fisher’s exact test was for significance testing and was calculated to compare all women with cervical cancer, HSIL, LSIL to women without lesion, regardless of HIV status. nd, not determined.

HPV genotypes detected in the eight different groups as classified by HIV and cytohistologic diagnosis of cervical lesions and cancer are shown in **Figure 3**. Overall, infections with HR-HPV types and in particular HPV16 predominated regardless of cytohistological diagnoses and HIV status as compared to low-risk HPV types (**Figure 3**).

### HPV Genotyping Analyses in Women Without Lesions

Two hundred twenty-eight HIV− and 198 HIV+ women without cervical lesions were randomly selected and subjected to HPV genotyping. Within these, HIV+ women were more frequently infected with HPV (64 *versus* 36%, p<0.0001) and—if HPV infected—had a higher proportion of multiple HPV types (64 *versus* 40%, p=0.0006) than HIV− women. Women up to 30 years of age had the highest HPV infection rates with 49 and 76% for HIV− (n=47) and HIV+ women (n=50), respectively, compared to women above 30 years (p<0.0001, chi-square test); of note, 23% (HIV−) and 63% (HIV+) women up to 30 years of age were infected with two or more HPV types. Rates of single- and multiple-type infections declined with age up to the age of 50 years; of all age groups, 41–50-year-old women had the lowest rates of HPV infections with only 31 and 54% of HIV− and HIV+ women being HPV infected, respectively. Similarly, the rate of HIV− and HIV+ women with two or more HPV types was decreased to 8 and 29% in this age group, respectively. There was a trend of higher rates of HPV infections in HIV− women above 50 years, compared to 31–40 and 41–50 year age groups (p=0.22); 8 of 34 HIV− women above 50 years (24%) had prevalent infection with HPV16, compared with only 6 of 61 (10%) in the 41–50 year group (p=0.13). In summary, in this univariate analysis of women without cervical lesions, younger age and HIV infection were associated with higher frequencies of HPV infections. ART− HIV^+^ women (n= 49) had slightly higher HPV infection rates compared to ART+ women (n=136, 73 *versus* 60% infected, p=0.09). There were no statistical differences in the number of HPV types detected per women between ART+ and ART− women without lesions.

HIV+ women without lesion were more frequently infected with a potentially carcinogenic HR-HPV type as compared to HIV− women (55 *vs* 31%, p<0.0001). HPV16 was detected in 14% of HIV− and 19% of HIV+ women without lesion. Infections with other HPV types were rarely detected (<5% prevalence per type) in HIV− women and were most often caused by the HPV45, 35, 33, each with prevalence rates of 4–5%, followed by HPV51 (3%). In HIV+ women without lesions, HR-HPV types 51, 45, 35, 66, 52, and 58 and 18 and the low-risk HPV types, HPV61, 62, and 68, all occurred at rates of 5 to 10%.

### HPV Genotyping Analyses in Women With Low-Grade Intraepithelial Lesions and/or CIN1

HIV− (n=13) and HIV+ women (n=61) with LSIL were subjected to HPV genotyping analyses. Fifty-three percent of HIV− and 97% of HIV+ women with LSIL had detectable HPV infections, and almost all these women were infected with at least one HR-HPV type. HPV16 was the most frequently detected HPV genotype in these women with infection prevalence of 32 and 41% for HIV+ and HIV− women, respectively. Thirty-one percent and 52% of HIV− and HIV+ women were infected with either HPV16, 18, and/or 45, which were found to be the most carcinogenic HPV types in our study. Besides HPV16, HPV35 (n=3) and 51 (n=2) infections were most frequent. Five of seven subjects had infections with more than one HR-HPV type. LSIL-positive women living with HIV were characterized by a high diversity of infecting HPV types; HPV51 was detected with second highest infection prevalence (20%), followed by HPV58, 35 (both above 15%) and 18, 33, 45, as well as some low-risk types as shown in **Figure 3**. More than one HPV type was detected in 70% of HIV+ women with LSIL regardless of ART status (p=1.0). When comparing women with LSIL to women without cervical lesions, the following HPV types were significantly associated with LSIL: HPV16 (p<0.0001), HPV51 and HPV73 (both p=0.001), HPV31 (p=0.004), HPV55 (p=0.006), HPV35 and HPV58 (p=0.008), HPV26 and HPV33 (p=0.02), and HPV18 (p=0.047).

In LSIL+ women living with HIV, the prevalence of clinically most relevant HR-HPV types differed by age groups; while women up to 30 years of age (n=18) had similar frequencies for HPV16, 18, 35, and 45 ranging from 18 to 28%, the relative frequency of HPV16^+^ LSIL findings increased to 48% (14/29) and 46% (6 of 13) in the age groups 31–40 and 41–50 years, respectively, whereas the frequency of HPV18 (3 and 15%), HPV35 (10 and 8%), and HPV 45 (3 and 8%) decreased in these age groups, which is consistent with a higher persistence of HPV16 infections as compared to the other clinically relevant HR-HPVs.

### HPV Genotyping Analyses in Women With High-Grade Intraepithelial Lesions and/or CIN2+

HPV infection patterns in women with HSIL were similar to what was observed in women with LSIL; 71% of HIV− (n=14) and 90% of HIV+ women (n=52) had detectable HPV infections. The vast majority of these infections were associated with one or more HR-HPV types. With 29% prevalence for both HIV infection strata, HPV16 was the most frequent type. Overall, 67% of HIV+ women with HSIL were infected with multiple HPV types, a higher proportion as compared to their HIV− counterparts (p=0.014). HSIL findings in HIV+ women were associated with a broad spectrum of HR-HPV types. Particularly high infection frequencies were found for HPV35 (26%), followed by HPV18, 33, 45, and 58 (both above 15%) and five additional HPV types occurring in >10% of women. There was no significant difference in the number of HPV types detected in ART+ (n=40) and ART− (n=9) HIV+ subjects with high-grade lesions (p=0.23). Within HSIL women living with HIV, HPV16 infection frequencies declined with age from 44% (4 of 9) in the <30-year-olds, to 27% (7 of 27) and 23% (3 of 13) in the 31–40 and 41–50 year age groups, respectively, whereas for other highly clinically relevant HR-HPV types, prevalence increased with age as follows in these same age groups: HPV18 occurred at 0% (0 of 9, <30 years), 19% (5 of 26, 31–40 years), and 23% (3 of 13, 41–50 years). HPV35 occurred at 22% (2 of 9), 23% (6 of 26), and 46% (6 of 13). HPV45 occurred at 11% (1 of 9), 7% (2 of 26), and 23% (3 of 13).

When comparing all women with HSIL to all women without cervical lesions, 13 HPV genotypes were associated with HSIL, with statistically significant results: HPV35 (p<0.0001), HPV33 (p=0.0006), HPV18 (p=0.002), HPV81 (p=0.002), HPV53 (p=0.004), HPV6 (p=0.005), HPV CP6108 (p=0.008), HPV58 (p=0.009), HPV16 (p=0.01), HPV45 (p=0.01), HPV73 (p=0.01), HPV62 (p=0.02), HPV39 (p=0.04).

Together our results so far show substantially increased prevalence rates of HPV and HR-HPV infection in HIV^+^ women regardless of lesion status, despite most of these being on efficient ART treatment. While ART suppressed HIV viremia in most of these women, it was not associated with a significant reduction in HR-HPV prevalence. LSILs and HSILs were often associated with diverse and multiple HR-HPV type infections in HIV+ women. However, the high prevalence of multiple HPV infections in these women did not allow to conclude whether detected HPV types caused HSIL with risk of further progression to cancer or were often just cohabitating. The decreasing prevalence of HPV16+ HSIL with age and high prevalence of HPV16+ cancers in women living with HIV, often at relatively young age, is consistent with the higher carcinogenic potential of HPV16, whereas HSILs associated with HPV35 may persist but not progress frequently, consistent with the high number of HV35+ HSILs in women living with HIV between 30 and 50 years of age.

### HPV16, 18, and 45 Potentially Cause the Vast Majority Cervical Cancers Regardless of HIV Infection and ART Status

**Figure 4:**
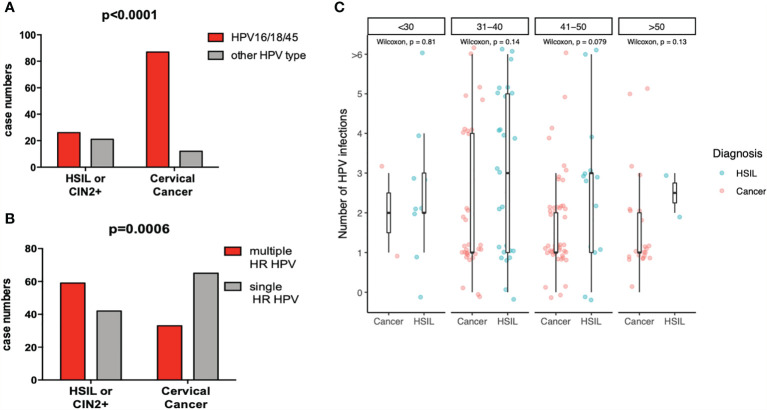
Decreased HPV type diversity and shifting HPV genotype distribution in HIV+ women with cervical cancer. **(A)** shows the number of subjects with HSIL and cervical cancer cases that were associated with HPV16, 18, or HPV45 infection (red bars) *versus* those that were not (gray bar) and **(B)** those associated with multiple HR-HPV types *versus* single HR-HPV types. Indicated p-values were calculated using the two-sided Fisher’s exact test. **(C)** shows the number of HPV types detected in HIV^+^ women with cervical cancer or HSIL in different age groups. Statistical analyses were performed by Wilcoxon test.

We originally hypothesized that higher proportions of none-16/18 HR-HPV types, which are frequently associated with squamous intraepithelial lesions in women living with HIV, also cause CC more frequently in HIV+ than in HIV− women. To address this hypothesis, 129 HIV− and 107 HIV+ women with CC were subjected to HPV genotyping analyses. HPV16 (52 *versus* 42%), 18 (22 *versus* 20%), and 45 (14% for both) occurred with similar prevalence rates in HIV+ and HIV− cancer cases, respectively (**Figure 3**). In contrast to our study hypothesis, the composition of HR-HPV types in cancers was overall comparable between HIV+ and HIV− women. When combining HPV16, 18, and 45, those accounted for the vast majority of HPV+ cancer cases regardless of HIV status, with 85% of HIV+ (87 of 99) and 84% of HIV− (92 of 110) (**Figure 4**). In contrast to HIV-associated cancer cases, only 54% (26 of 48) of HIV-associated HSIL cases were associated with 16/18/45-HPV type infections (p<0.0001, **Figure 4A**). Further multiple HR-HPV types were detected in 59 of 101 HIV+ women with intraepithelial cervical lesions, but in only 33 of 99 HIV+ cancer cases (p=0.0006, **Figure 4B**, HPV+ cases only). Similarly, in women living with HIV, the median number of HPV types was reduced in cases of cancer compared to women with cervical lesions (**Figure 4B**), and this may relate. Together these data show that the number of HR-HPV types of cancer cases was significantly contracted in HIV+ women with CC women with single HR-type infections causing the majority HIV+ cancer cases; HPV16 (n=36), HPV18 (n=14), and HPV45 (n=7) and HPV35 (n=4), HPV31 (n=2), HPV52 and 59 (both n=1) were detected as a single-genotype infection in HIV+ cancer cases.

While most cancers were squamous cervical cancers, 12 cases were diagnosed as adenocarcinomas. In five of these, no HR-HPV type was detected. HPV18 was found in five HIV− women and was the most frequently detected HR-HPV type in adenocarcinoma cases. HPV16 was found in two adenocarcinoma cases in combination with HPV18 or HPV39. HPV45 was detected as a single infection in one HIV+ adenocarcinoma case.

In summary, HPV16 was by far the most frequent HPV type causing CC, followed by HPV18 and 45 regardless of HIV status. In HIV+ CC cases, HPV type diversity was reduced, and distribution pattern shifted towards few cancer-causing types when compared to high- and low-grade intraepithelial cervical lesions in women living with HIV. While other HR-HPV types, such as HPV35 and 58, were disproportionally increased in HSIL, these were infrequently detected in cancer cases regardless of HIV infection (see below). Hence, such types most probably have a high potential to cause “precancerous” lesions in women living with HIV, but with relatively little risk of progression. Instead, lesions caused by such types may persist for a very long time without progression or eventually be cleared even in many women living with HIV. Interestingly, there was no difference in the age distribution between HIV^+^ women with CC associated with HPV16 (n=55) *versus* CC associated with non-HPV16 types, suggesting a comparable pace of cancerogenesis between HPV16 and HPV18/45+ precancerous lesions to cancer.

## Discussion

The 2H study is among the largest prospective studies to date with the aim to dissect mechanisms underlying the HIV-associated risk-increase for CC progression in a setting of high endemicity for both infections. The study screened more than 2,000 women for cervical cancer and precancerous lesions who were then classified into four groups delineated by HIV and cytohistological diagnosis. We found (1) that the vast majority of women who received a diagnosis of LSIL and HSIL were HIV positive; (2) that any diagnoses of cervical lesion and cancer, as well as HIV infection and low CD4 counts, were identified as independently associated with infection with one or more HPV types and with HR-HPVs, which accounted for the vast majority of these infections; and (3) that—against our original hypothesis—HPV16, 18, and also 45 infections accounted for roughly 85% of HPV+ cancers in women regardless of their HIV status, whereas more diverse HR-HPV type infections were associated with HSIL. Importantly, age was associated with a reduction of HPV types in women living with HIV, suggesting that most HR-HPV infections are eventually being cleared despite HIV.

There is a broad consensus that molecular diagnoses of HR-HPV infection should be incorporated into standard CC screening procedures to identify and monitor risk for HPV disease progression (23) (S3 Guidelines Germany). Women above 30 years with persistent HR-HPV infection have a high risk to develop high-grade precancerous cervical lesions (24, 25), and a high proportion of these develop cervical cancer. One major problem to incorporate molecular diagnoses in a meaningful way to CC screening procedures tailored to women living with HIV is the high prevalence and diversity of HR-HPV infections in these women with and without precancerous lesions (16, 19–21). Some of these reports implied that *less virulent*, non-HPV16/18 HR-HPV types are responsible for a greater proportion of CC cases as compared to HIV negative women (15, 16, 20, 21). Our results are consistent with these previously published data for women living with HIV showing a high diversity of HR-HPV types in these women but argue against the hypothesis that HIV substantially alters the relative carcinogenicity of less virulent HR-HPV types as reported previously (21). HIV-associated depletion of HR HPV oncogene-specific T cell responses in coinfected women may potentially contribute to the increased HPV prevalence and persistence rates (Mbuya et al., 2021, in revision).

HPV35, which is not included in the Gardasil9 vaccine and closely related to HPV16, is a genotype of specific interest. A recent study suggested a strong link between HPV35 and cervical carcinogenesis, particularly in women of African ancestry (26). In our study, HPV35 was the most prevalent in HSILs and detected in 11% (HIV+) and 7% (HIV−) cancer cases. However, only few of these cancers contained HPV35 alone. Instead, 67 and 56% of HPV35+ cancers in women living with and without HIV contained additional HR-HPV types, most often HPV types 16, 18, and/or 45 (as well as others). HPV35+ cancers may have been caused by these “very high risk” types, and we consider this likely in most of these cases. Indeed, roughly 80% of HPV35+ HSILs also contained other HR-HPV types in our study. Hence, the cancerogenic potential of HPV35 is well probably below that of HPV16, 18, and 45 also in the studied sub-Saharan African populations. One possible explanation for this frequent “co-habitation” in cancer and HSIL cases is that HPV35 may benefit from a precancerous or cancerous microenvironment caused by other HR-HPV infections.

Our results therefore suggest that infections with HPV16, 18, and 45, which cause the greatest risk for women in Africa, are by far the most dangerous types also in HIV+ women to develop CC. Indeed, the HR-HPV genotype distribution pattern in HIV+ women observed in our study was almost identical to those reported in a recent meta-analyses (27) that included data from above 700 CC and almost 400 HSIL cases. Further, in our study, HPV58 and 35 were significantly associated with and frequently detected in HSIL cases but comparatively infrequent in HIV+ CC cases, which again is consistent with the data reported previously (27). Other HR-HPV types were often detected in HIV+ CC cases as compared to HIV− cancer cases but were primarily accounted for by multiple HPV infections, similar to what we highlighted for HPV35 in our study. Together these studies provide solid evidence that infections with HR-HPV types other than HPV16/18 and 45 often do not progress to cancer in HIV+ women, despite their potential to induce HSIL or cohabitate with HR-HPV types. This finding is important because HPV16/18 vaccination provides some cross-protection also from HPV45 infection (as well as HPV31 and 33) and associated CC progression (28). It is therefore most important to address whether protective efficiency of HPV vaccination is reduced by subsequent HIV infection. If not, mass vaccinations even using standard HPV16/18 vaccination should end this CC epidemic regardless of HIV control measures.

CC screening algorithms should be optimized and, if necessary, tailored to the special needs of women in resource-poor settings. The paucity of trained pathologists makes diagnosis of precancerous lesions difficult to impossible in many resource-poor settings of SSA. Further, Pap smear and histology-based diagnosis of intraepithelial lesions is extremely time-consuming and basically impossible to implement and scale up in most settings in SSA. Molecular HR-HPV tests—particularly assays based on RT-PCR—have certain advantages over screening by cyto-histology; they are comparatively easy to implement, standardize, highly specific to detect HR-HPV infections, and can in principle be upscaled to high throughput at the point-of-care. The problem of this approach, particularly in HIV+ women, is many HR-HPV genotyping tests do not differentiate between the 13 HR-HPVs. As infection with diverse HR-HPV types is so common in HIV+ women, molecular diagnostic tests generally should better differentiate between HPV types with high, medium, and very low risk of carcinogenesis. Type-specific diagnosis will much more accurately link HPV diagnosis with risk of cancer progression regardless of HIV status. Further, only type-specific diagnostic methods can diagnose truly persistent HR-HPV infections as a cause of disease progression (24). In SSA settings, molecular diagnostic approaches should therefore particularly focus on HPV16, 18, or 45 women for advanced diagnostic and therapeutic workup, e.g., in centralized facilities.

One major limitation of this study was the numbers reported for HPV52 may be too low, because the method used did not allow accurate detection of this HPV type, particularly in our study characterized by many multiple HR-HPV infections. The study also did not include enough patients with a diagnosis of adenocarcinoma to draw any definite conclusions. However, the fact that HPV18 infections accounted for most of the HPV+ adenocarcinomas and that five out of 12 adenocarcinomas were HPV negative is of interest. Possibly, several of these patients did not have adenocarcinoma of the cervix but of adenocarcinoma of the endometrium with involvement of the cervix or metastatic disease from other sites (especially gastrointestinal sites). To differentiate between cervical and endometrial adenocarcinoma using HE staining alone can be difficult. p16 and E2 immunostaining would have been helpful but was not performed. Similarly, in a significant fraction (16 of 129) of non-HIV-associated squamous cervical cancers, no HPV type was detected and hence etiology remained unclear.

In conclusion, our data show that there is a contraction of HR-HPV diversity during carcinogenesis with HPV16, 18, and 45 causing the vast majority of CC regardless of HIV infection status, whereas a more diverse spectrum of HR-HPVs often causes cervical intraepithelial lesions. Diagnostic strategies in SSA settings would benefit from incorporating molecular diagnosis of individual HR-HPV types to identify and target women infected with the clinically most relevant HR-HPV+ infection instead of diagnoses of HR-HPV infections without further differentiation.

## Data Availability Statement

The raw data supporting the conclusions of this article will be made available by the authors, without undue reservation.

## Ethics Statement

The studies involving human participants were reviewed and approved by Mbeya Medical Research and Ethics Review Committee (MRH/R.10/8/Vol. VI/107). The patients/participants provided their written informed consent to participate in this study.

## Author Contributions

RM, TL, LM, MP, BM, MS, LH, MJ, and JF contributed to the clinical cohort work. LT and NS contributed to cytohistological diagnosis of cervical lesions and cancer. MG contributed to statistical data analyses and drafting of figures. NC and ES contributed to data management. WiM, WoM, AM, JM, and AB contributed to HPV genotyping analyses. RM contributed to analyses of other clinical data. MC coordinated laboratory workup of study samples. RL, LM, and MH provided senior advice and study-specific capacity building and contributed to manuscript writing. AK and CG conceived and coordinated the 2H study and secured funding. All authors contributed to manuscript writing and concur with the manuscript submission.

## Funding

The 2H study received funding from the Deutsche Forschungsgemeinschaft (reference number; 2128/2-1 and 2128/2-2, Project number 620615) and DZIF (AINVAC B). RM was supported by DELTAS Africa Initiative grant # DEL-15-011 to THRiVE-2. MG acknowledges the support from the Joachim Herz Foundation through the Add-on Fellowship for Interdisciplinary Science.

## Conflict of Interest

The authors declare that the research was conducted in the absence of any commercial or financial relationships that could be construed as a potential conflict of interest.

## Publisher’s Note

All claims expressed in this article are solely those of the authors and do not necessarily represent those of their affiliated organizations, or those of the publisher, the editors and the reviewers. Any product that may be evaluated in this article, or claim that may be made by its manufacturer, is not guaranteed or endorsed by the publisher.
