# Supplementary material for: HPV Type Distribution in HIV Positive and Negative Women With or Without Cervical Dysplasia or Cancer in East Africa
Source: Front Oncol. 2021 Nov 30;11:763717. doi: 10.3389/fonc.2021.763717 (PMC8669270; doi:10.3389/fonc.2021.763717)
Supplement: Supplementary file 1 [file Image_1.pdf]

Supplementary figure 1_Mcharo et al. 2021

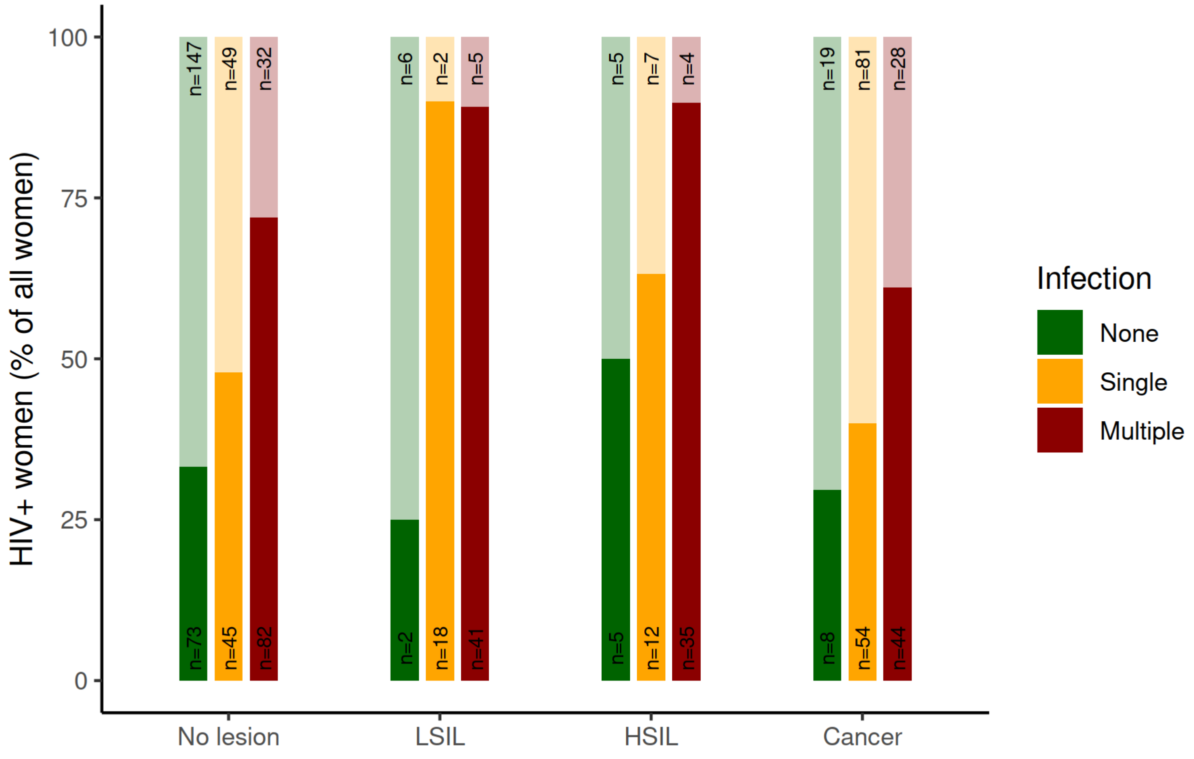

Distribution of single and multiple HPV infections among HIV+ and HIV- women with no lesion, LSIL, HSIL and Cancer. Shown is the percentage of HIV+ women (as percent of all women, y-axis) within the disease categories No Lesion, LSIL, HSIL and Cancer (x-axis), who are not HPV infected (green bars), who are infected with a single HPV type (orange bars) and those with multiple HPV types (red bars). The transparent counter-part of each bar to 100% in the transparency shows indicated the proportion of HIV- women in each group.
